# Characterization of Collagen Binding Activity of *Clostridium perfringens* Strains Isolated from Broiler Chickens

**DOI:** 10.3390/pathogens12060778

**Published:** 2023-05-30

**Authors:** Zhifeng Sun, Mingmin Lu, Hyun Lillehoj, Youngsub Lee, Doyun Goo, Baohong Yuan, Xianghe Yan, Charles Li

**Affiliations:** 1 Animal Bioscience and Biotechnology Laboratory, Beltsville Agricultural Research Center, Agricultural Research Service, US Department of Agriculture, Beltsville, MD 20705, USA; 2 Environment Microbial and Food Safety Laboratory, Beltsville Agricultural Research Center, Agricultural Research Service, US Department of Agriculture, Beltsville, MD 20705, USA

**Keywords:** *Clostridium perfringens*, collagen binding, collagen adhesin protein, *cnaA*, *netB*, *tpeL*

## Abstract

*Clostridium perfringens* is the etiological agent for necrotic enteritis (NE) in broiler chickens, which causes a substantial economic loss of an estimated USD 6 billion annually in the global poultry industry. Collagen adhesion is involved in the NE pathogenesis in poultry. In this study, the binding capabilities of chicken *C. perfringens* isolates of various genetic backgrounds (*netB^−^tpeL^−^*, *netB^+^tpeL^−^*, *netB^+^tpeL^+^*) to collagen types I–V and gelatin were examined, and the putative adhesin protein *cnaA* gene was investigated at the genomic level. In total, 28 *C. perfringens* strains from healthy and NE-inflicted sick chickens were examined. The results on collagen adhesin-encoding gene *cnaA* by the quantitative-PCR results indicated that *netB^−^tpeL^−^* isolates had much lower copies of the detectable *cnaA* gene than *netB^+^* isolates (10 *netB^+^tpeL^−^ isolates*, 5 *netB^+^tpeL^+^* isolates). Most of the virulent *C. perfringens* isolates demonstrated collagen-binding abilities to types I–II and IV–V, while some strains showed weak or no binding to collagen type III and gelatin. However, the *netB^+^tpeL^+^* isolates showed significantly higher binding capabilities to collagen III than *netB^−^tpeL^−^* and *netB^+^tpeL^−^* isolates. The data in this study suggest that the collagen-binding capability of clinical *C. perfringens* isolates correlates well with their NE pathogenicity levels, especially for *C. perfringens* isolates carrying genes encoding crucial virulence factors and virulence-associated factors such as *netB, cnaA*, and *tpeL*. These results indicate that the presence of the *cnaA* gene may be correlated with *C. perfringens* virulence (particularly for *netB^+^* isolates).

## 1. Introduction

*Clostridium perfringens* is an anaerobic Gram-positive rod-shaped bacterium, and its type A and G isolates are the key etiologic factor contributing to necrotic enteritis (NE) in broiler chickens [1]. NE is characterized by sudden death (clinical form) or underperformance in growth and feed conversion (subclinical form), which could inflict substantial economic damage with an estimated annual loss of USD 6 billion to the global poultry industry [2,3]. NE is a complex and multi-factorial disease [3]. The *β*-pore-forming toxin netB is found to be an essential contributing factor to the virulence of pathogenic *C. perfringens* isolates [4,5,6]. Disease severity can be enhanced by multiple factors, such as using highly pathogenic *netB^+^tpeL^+^* isolates expressing the largest TpeL toxin (a size of ~205 kDa), predisposition to coccidiosis, and nutritional manipulation with the use of cereals rich in non-starch polysaccharides or animal proteins [3,7].

Bacterial colonization is the first step for infection and plays a critical role in the normal development, differentiation, function, and regulation of the intestinal mucosal immune system [8]. Bacteria have evolved various mechanisms to adhere to the surface of organs in contact with the intestine, such as adhesin, pili, fimbriae, biofilm, and lipopolysaccharides [9]. It is suggested that the strong capability of *C. perfringens* adherence to extracellular matrix molecules promotes virulence in the induction of NE [10]. Furthermore, the adherent capabilities of *C. perfringens* isolates to collagens may be positively associated with the pathogenesis of avian NE [11,12]. The putative collagen gene *cnaA* is located within pathogenicity locus VR-10B mainly in *netB*-positive strains [13], and the mutation of *cnaA* has abolished NE pathogenesis in an animal model and reduced the capability of bacterial colonization in the chicken intestinal mucosa [11]. In this study, we characterized the capability of 28 *C. perfringens* isolates of laboratory collections in adherence to several collagens (I, II, III, IV, and V) and gelatins and screened the *cnaA* gene in bacteria with a polymerase chain reaction (PCR) and quantitative-PCR (qPCR).

## 2. Materials and Methods

### 2.1. Bacterial Strains and Growth Conditions

Twenty-eight isolates of *C. perfringens* were used in this study. N10 and N11 were isolated from healthy chickens, while most others came from collections of NE-afflicted chickens [14]: nine *netB^−^tpeL^−^* isolates, fourteen *netB^+^tpeL^−^* isolates, and five *netB^+^tpeL^+^* isolates. The isolates carrying the *tpeL* gene may induce enhanced NE disease severity. All strains were characterized for *cnaA* gene by PCR and qPCR. Of all 28 *C. perfringens* isolates, some have been characterized for NE induction in our NE disease model (See Table 1).

All *C. perfringens* strain collections were maintained as stock cultures in 25% glycerol and stored in a –80 °C freezer in the laboratory [15,16,17]. *C. perfringens* strains were grown anaerobically at 37 °C in chopped meat glucose (CMG) medium (Anaerobe Systems, Morgan Hill, CA, USA), and then in BYC medium containing 3.7% brain heart infusion medium (BHI Broth, RPI, Research Products International, Mt. Prospect, IL, USA), 0.5% yeast extract (Fisher Scientific, Hampton, NH, USA), 0.05% L-cysteine (Sigma-Aldrich, St. Louis, MO, USA), and 0.1% sodium thioglycolate (Sigma-Aldrich). The bacteria were incubated at 37 °C under anaerobic conditions (AnaerPack^®^ System, MGC, Mitsubishi Gas Chemical Co., Inc., Tokyo, Japan) overnight.

**Table 1:** Sources and background information of *Clostridium perfringens* collections.

Strain	Source	*netB*	*tpeL*	Reference
13	Soil	−	−	This study
CP1	Field NE	−	−	[16]
CP2	Field NE	−	−	[16]
CP15	Field NE	−	−	[16]
CP23	Field NE	−	−	[16]
JGS	Field NE	−	−	[16]
LLY_N10	Healthy chickens	−	−	[16]
LLY_N11	Healthy chickens	−	−	[16]
SM101	Food poisoning	−	−	[17]
C11	Field NE	+	±	[15]
tpeL15	Field NE	+	+	[15]
tpeL17	Field NE	+	+	[15]
tpeL18	Field NE	+	+	[15]
tpeL19	Field NE	+	+	[15]
C1	Field NE	+	−	[15]
C2	Field NE	+	−	[15]
C3	Field NE	+	−	[15]
C5	Field NE	+	−	[15]
C6	Field NE	+	−	[15]
C7	Field NE	+	−	[15]
C8	Field NE	+	−	[15]
C9	Field NE	+	−	[15]
C10	Field NE	+	−	[15]
C12	Field NE	+	−	[15]
C13	Field NE	+	−	This study
C14	Field NE	+	−	[15]
C16	Field NE	+	−	[15]
Del1	Field NE	+	−	[16]

Note: “−” = negative in PCR, “+” = postive in PCR, “±” = weakly positive in PCR.

### 2.2. Preparation of Genomic DNA from C. perfringens

Five mL of BHI Broth was inoculated with each strain of *C. perfringens* and incubated at 37 °C under anaerobic conditions overnight. An amount of 1.5 mL of the culture was centrifuged at 8000× *g* for 2 min in a microcentrifuge, and the pellet was collected. Then, 100 μL of 200 mg/mL lysozyme was added to each tube, mixed thoroughly, and incubated at 37 °C for 30 min. Samples were vortexed well, and then incubated at 37 °C for an additional 15 min; 467 µL of TE buffer was added, and the pellet was resuspended by repeated pipetting. Then, 30 µL of 10% SDS and 3 µL of 20 mg/mL proteinase K (Sigma-Aldrich) were added to give a final concentration of 100 µg/mL proteinase K in 0.5% SDS. Samples were mixed thoroughly and incubated at 37 °C for 1 h. Then, 100 µL of 5 M NaCl was added and mixed thoroughly, followed by adding 80 µL of cetyltrimethylammonium bromide (CTAB, Sigma-Aldrich) /NaCl solution for a 10 min incubation at 65 °C. An approximately equal volume (0.7 to 0.8 mL) of chloroform/isoamyl alcohol (Sigma-Aldrich) was added and mixed thoroughly, and samples were centrifuged for 5 min. Aqueous and viscous supernatants were transferred to new tubes. An equal volume of phenol/chloroform/isoamyl alcohol (Sigma-Aldrich) was then added, extracted thoroughly, and centrifuged for 5 min. The supernatant was transferred to a fresh tube, and a volume of 0.6 of isopropanol was added to precipitate the nucleic acids. DNA was washed with 70% ethanol to remove residual CTAB, and DNA was centrifuged for 5 min at room temperature. The supernatant was carefully removed, and the pellet was briefly air-dried. The pellet was redissolved in 100 µL of TE buffer.

### 2.3. PCR for Detection of CnaA

All *C. perfringens* strains were screened for the presence of *cnaA* using PCR. Primers were designed using the *cnaA* gene of strain EHE-NE 18 (Accession number KT749987.1) (Table 2). Primer sequences were as follows: forward primer 5′-GGTGGATGGGCAACATTTAC-3′, reverse primer 5′-CCTTGCTTGGATTCACCAGT-3′, with an expected product size of 220 bp. PCR was performed using GoTaq green master mix (Promega, Madison, WI, USA). PCR conditions were used as follows: 95 °C for 3 min; followed by 35 repeats of 95 °C for 30 s, 55 °C for 30 s, and 72 °C for 3 min; followed by 72 °C for 5 min.

### 2.4. qPCR for Detection of cnaA

Genomic DNA (gDNA) was assessed using a NanoDrop spectrophotometer (NanoDrop One; Thermo Fisher Scientific, Waltham, MA, USA) at 260/280 and 260/230 nm to measure its concentrations and to check its purity. Each sample of gDNA was diluted to a concentration of 50 ng/µL in RNase-free water (Thermo Fisher Scientific), and followed by qPCR analysis with 5 μL of cDNA using SYBR Green qPCR Master Mix (PowerTrack, Applied Biosystems, Waltham, MA, USA) in triplicates using Applied Biosystems QuantStudio 3 Real-Time PCR Systems (Life Technologies, Carlsbad, CA, USA). The gDNA was analyzed with the following PCR conditions: denaturation at 95 °C for 10 min, followed by amplification at 56 °C for 1 min for 40 cycles. The oligonucleotide primer sequences used for 16S rRNA and *cnaA* genes are listed in Table 2. The relative expression level of each gDNA was normalized to the control gene 16S rRNA using the 2^−ΔΔCt^ method [15].

### 2.5. Adhesion Assay

Bacterial adherence to five types of collagens and one gelatin was conducted as previously described and repeated in triplicates [12]. Collagens (types I, II, III, IV, and V) were all purchased from Sigma: collagen I from rat tail; collagen II from bovine nasal septum; collagen III from human placenta and Bornstein and Traub type III (Sigma Type X); collagens IV and V both from human placenta and gelatin (gelatin from porcine skin, gel strength 300, type A). Nunclon Delta-Treated, Flat-Bottom Microplates (Thermo Fisher Scientific) were coated with collagens (1 µg/well for collagens III and V; 2 µg/well for collagens I, II, and gelatin; and 0.031 µg/well for collagen IV) in 50 µL of phosphate-buffered saline (PBS, Quality Biological Inc., Gaithersburg, MD, USA) overnight at 4 °C and blocked using 200 μL of PBS plus 0.5% (*w*/*v*) bovine serum albumin (BSA) (Sigma-Aldrich) (PBSA) for 2 h at 4 °C and rinsed three times with 200 μL of PBSA. 

Single *C. perfringens* colonies were suspended in 100 μL of sterile PBSA, spread onto BHI-supplemented *C. perfringens*-specific nutrient agar plates, and incubated overnight to produce a confluent layer. Bacteria were harvested from the agar surface in PBSA. Cells were pelleted by centrifugation at 3000× *g* for 2 min and washed three times with 5 mL of PBSA. The suspension was adjusted to an optical density (OD) of around 0.8 at 600 nm. Cells were added to wells in 50 μL aliquots and incubated at room temperature for 2 h with agitation, followed by rinsing three times with 200 μL of PBSA. *C. perfringens* were stained with 0.5% (*w*/*v*) crystal violet for 5 min. After rinsing three times with 200 μL of PBSA, 50 μL of solution of ethanol:acetone (1/1, *v*/*v*) was added to each well to destain adherent *C. perfringens*, and the OD value at 562 nm was measured. Wells incubated with bacteria but no collagen, and collagen-coated wells to which no bacteria were added, were used as blank and negative controls, respectively. Values from the adhesion assay were background-corrected by subtracting the blank value from the test sample absorbance values for that particular strain. Any values that were less than zero were adjusted to zero. All assays were repeated in biological triplicates.

### 2.6. Statistical Analysis

Statistical analysis was undertaken using GraphPad Prism 5 (GraphPad, San Diego, CA, USA). Mean treatment group values were compared using one-way analysis of variance followed by a Dunnett post hoc test. Differences were considered statistically significant at *p* ≤ 0.05. All the data were expressed as mean ± standard error of the mean.

## 3. Results

### 3.1. PCR for Detection of CnaA

**Figure 1:**
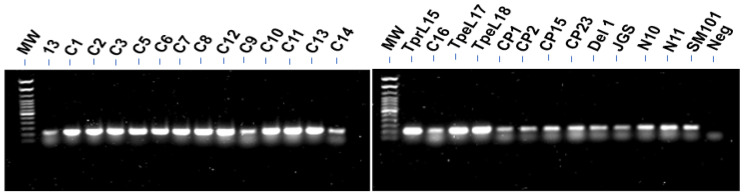
Detection of *cnaA* gene from *C. perfringens* isolates by PCR. Extracted genomic DNAs were applied as templates, and a pair of *cnaA* primers were used to amplify the *cnaA* gene. The PCR products were run on 1% agarose gels and imaged with the Chemi-Touch image analysis system. Neg = negative buffer control.

Fifteen of 28 *C. perfringens* strains tested showed thick bands for the *cnaA* gene (isolate tpeL19 not shown in Figure 1), among which all were *netB^+^* isolates, including five *netB^+^tpeL^+^* isolates (C11, tpeL15, tpeL17-19), while thirteen isolates had relatively thin bands for *cnaA* by PCR (Figure 1): strains 13, C9, C14, C16, CP1, CP2, CP15, CP23, Del1, JGS, N10, N11, and SM101, among which most were *netB^−^tpeL^−^*, and four *netB^+^tpeL^−^* isolates (C9, C14, C16, Del1). The PCR screening results for the *cnaA* gene within the genomes of these *C. perfringens* strains are summarized in Table 1.

### 3.2. Quantitative Polymerase Chain Reaction (qPCR) for cnaA Gene Detection

**Figure 2:**
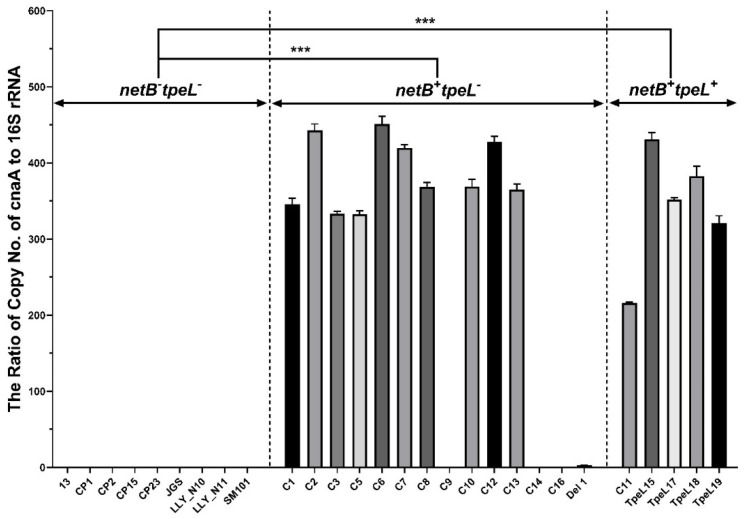
Detection of *cnaA* gene copies from *C. perfringens* isolates shown as the relative gene copies by qPCR. The gDNA was extracted, and the relative gene copy numbers of *cnaA* in real-time PCR were estimated by qPCR and normalized based on relative 16S rDNA levels. The data were analyzed by one-way ANOVA, and means were calculated using Duncan’s multiple range test. Each bar represents the mean ± SEM (*n* = 3). *** represents *p* ≤ 0.001.

Twenty-eight *C. perfringens* isolates were tested for *cnaA* presence by qPCR, among which 15 of them showed higher signals (high copy numbers of *cnaA*), and the remaining 13 of the isolates were negative (Figure 2), which is correlated well with the regular PCR result (Figure 1 and Table 3). No significant differences were found in the copy numbers of the cnaA gene between *netB^+^tpeL^+^* and *netB^+^tpeL^−^* (*p* > 0.05). However, significant differences were observed either between *netB^−^tpeL^−^* and *netB^+^tpeL^−^*, or between *netB^−^tpeL^−^* and *netB^+^tpeL^+^* (*p* < 0.001). Generally, there is a positive association between *cnaA* gene and *netB* gene expression except for a few samples (C5C8), which agrees with our previously published data on *netB* and *tpeL* gene expression [15].

### 3.3. Adhesion Assay

**Figure 3:**
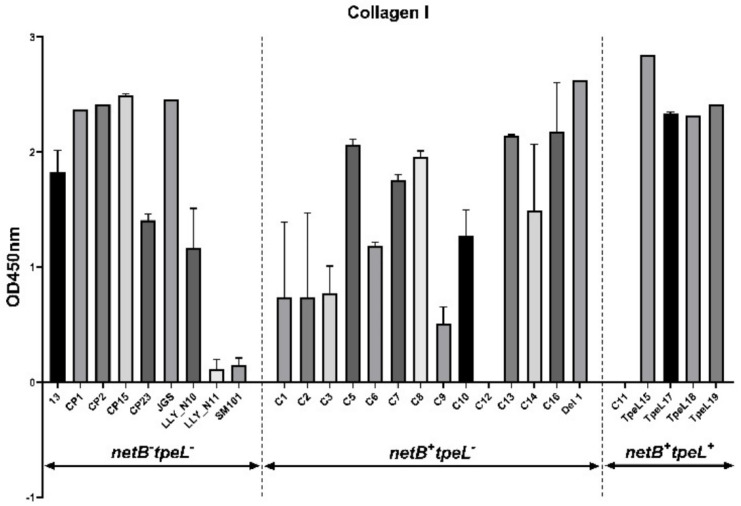

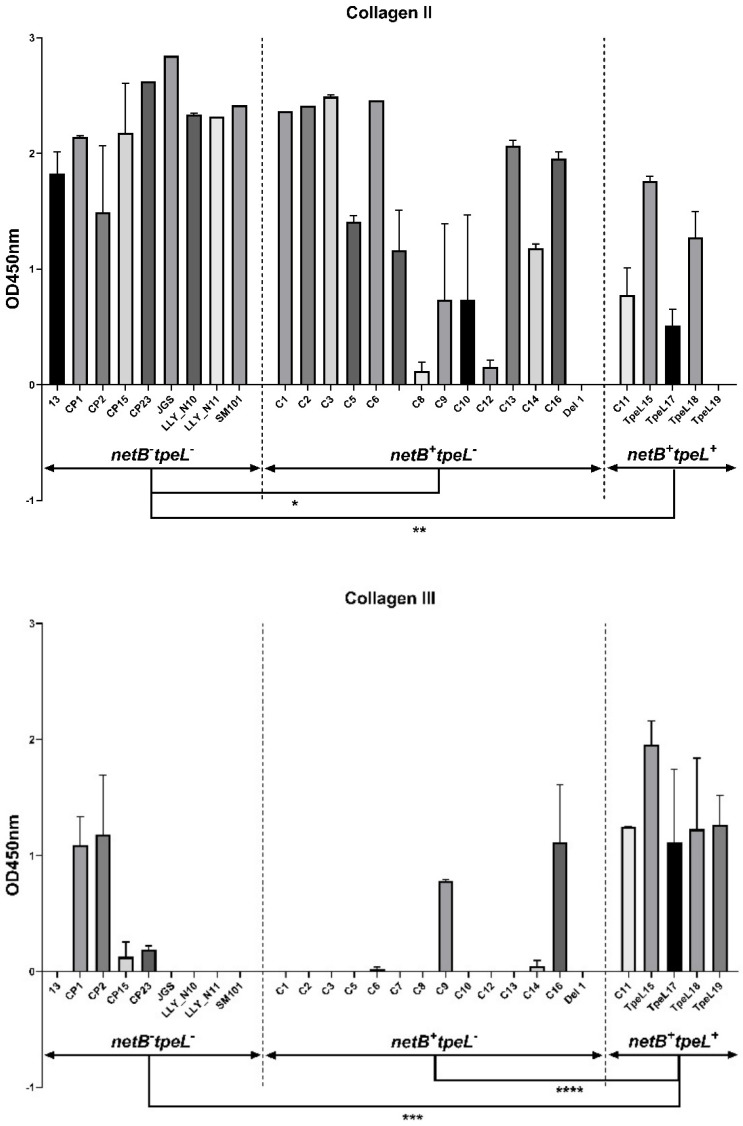

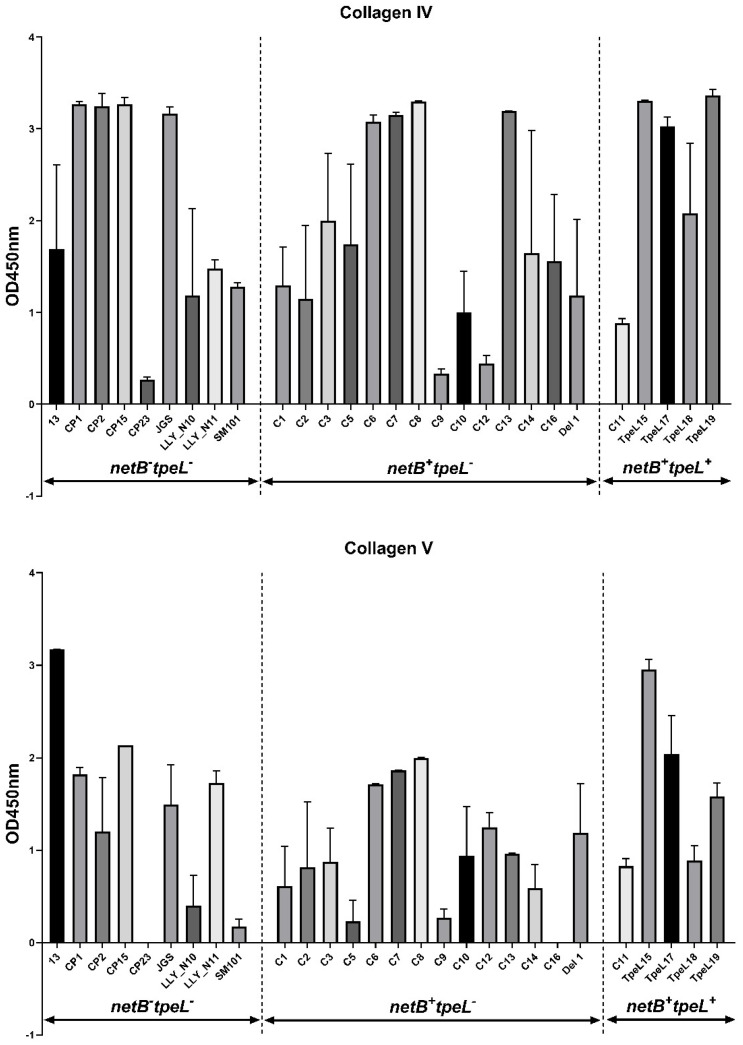

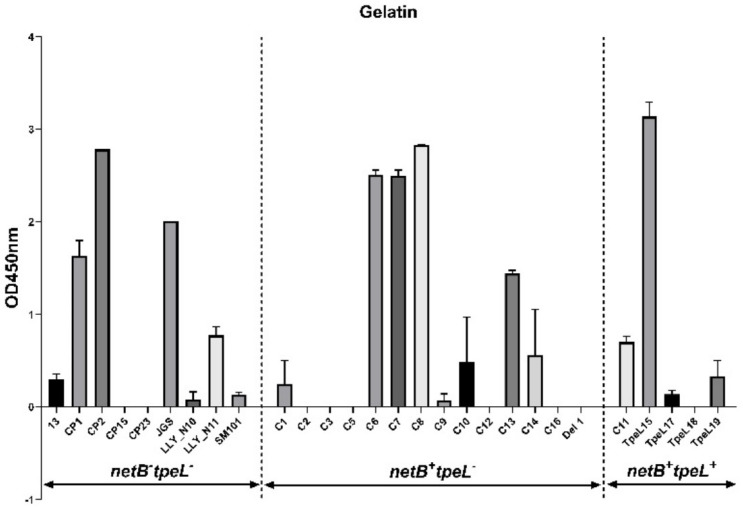
Adhesion assay of *C. perfringens* isolates to collagen types I, II, III, IV, and V, and gelatin. Collagens (types I, II, III, IV, and V) and gelatin were coated on Nunclon Delta-Treated, Flat-Bottom Microplates overnight at 4 °C and blocked using PBS plus 0.5% (*w*/*v*) bovine serum albumin. Bacterial cells were added to wells and incubated at room temperature for 2 h with agitation, followed by rinses. *C. perfringens* were stained with 0.5% (*w*/*v*) crystal violet for 5 min. After rinsing, solution of ethanol:acetone was added to each well to destain adherent *C. perfringens*, and the OD value at 562 nm was measured. All assays were repeated in triplicates. *, **, ***, **** marks represent statistically *p* ≤ 0.05, 0.01, 0.001, and 0.0001, respectively.

The 28 *C. perfringens* isolates were tested for their abilities to bind to collagens, and results show that *C. perfringens* isolates showed variable levels of binding to collagens. The majority of the virulent isolates were capable of binding well to collagen types I, II, IV, and V, while most of the isolates bound poorly to collagen type III and gelatin (Figure 3). The *netB^−^tpeL^−^* isolates showed the significantly stronger capabilities of binding to collagen type II than the *netB^+^tpeL^−^* (*p* < 0.05) and the *netB^+^tpeL^+^* isolates (*p* < 0.01). Interestingly, out of eight isolates binding potently to collagen type III, five isolates came from *netB^+^tpeL^+^* isolates (C11, tpeL15, tpeL17-19) (OD_562_ ≥ 1.0). The *netB^+^tpeL^+^* isolates demonstrated the significantly superior binding capabilities to collagen type III than either the *netB^−^tpeL^−^* (*p* < 0.001) or the *netB^+^tpeL^−^* isolates (*p* < 0.0001). The *netB^+^tpeL^+^* isolate tpeL15 bound firmly to the collagen types I~V and gelatin.

## 4. Discussion

*C. perfringens* is a complex pathogen, causing histotoxic infections, enteritis/enterocolitis, and enterotoxemias with this virulence versatility attributable mostly to its ability to produce various potent toxins [18], which results in the intestinal tissue damage associated with NE, exposure of extracellular matrix molecules (ECMM, such as collagen types I–III and V), and basement membrane or substances in the lamina propria (collagen type IV being the major component) [10]. Other than toxins, other *C. perfringens* virulence factors and their contribution to NE need to be better investigated. Furthermore, *C. perfringens* pathogenicity depends upon the colonization to persistently proliferate/survive in vivo, and this colonization process normally consists of nutrient acquisition for growth/survival (for example, by the production of sialidases NanI) and adherence [18]. In addition to sialidases, adhesins play important roles in the *C. perfringens* colonization stage for the pathogenesis of *C. perfringens* prior to secreting a panoply of toxins in the tissues.

It has been reported that the levels of virulence of *C. perfringens* strains are related to their binding abilities to collagen types II, IV, and V [10,12]. In the initial colonization stage, *C. perfringens* readily adheres to the damaged tissue where many extracellular matrix proteins, including abundant collagens and fibronectin (Fn) proteins, are presented in the damaged tissue [12]. *Eimeria* pre-exposure, which damages gut tissues and enhances the development of NE, acts as an essential predisposing factor in chickens [3,19].

Interaction between *C. perfringens*, tissue collagens, and cnaA as a pilus component of *C. perfringens* contributes to enteritis in chickens and pigs by promoting the adhesion to damaged intestinal tissue [10,12,20]. Inactivation of the pilus genes resulted in the inhibition of pilus production, highly reducing the capability of *C perfringens* to bind collagen and initiate disease [11,12,21]. It is also implied that fibronectin is a possible extracellular matrix glycoprotein that facilitates host–pathogen contact for *C. perfringens* to invade and colonize the gut [18,22,23]. In the present study, the majority of *C. perfringens* isolates came from cases of avian NE, and some of them, including the *netB^−^tpeL^−^* isolates, were capable of inducing NE when chickens were pre-exposed to coccidiosis. Within the virulent *netB^+^* isolates of *C. perfringens*, their abilities to adhere to collagen types I, II, IV, and V may be correlated with the presence of a putative collagen adhesin *cnaA* gene. Therefore, this study demonstrated a direct association between the virulence of *C. perfringens* strains and their abilities to adhere to immobilized collagens and between the virulence and the *cnaA* gene copies. Our results were in line with the observations obtained from other researchers [10,12]. Interestingly, most *C. perfringens* strains did not directly bind well to collagen type III or gelatin, although they bound to collagen types I, II, IV, or V. Possibly, the loss or reduced expression of fibronectin proteins on these isolates may impact the collagen binding ability. It has been reported that *C. perfringens* may firmly bind to collagens in the presence of fibronectin, therefore enhancing adherence and colonization [18]. This hypothesis merits further investigation. Mutations of three pilin structure subunits (*cnaA*, *fimA*, and *fimB)* have been reported to lead to the loss of filamentous structures; reduce the capabilities of *C. perfringens’s* binding to collagen types I, II, and IV; and result in attenuation of their pathogenicity in a chicken NE challenge model [21]. Understanding the roles of these virulence-associated factors in NE pathogenesis may help scientists to design optimal strategies to efficiently disrupt the colonization of *C. perfringens* during the early infection stage. Chickens immunized subcutaneously three times with either CnaA or FimB (another pilin structure subunit) showed significantly reduced NE lesions against pathogenic *C. perfringens* challenge [24]. In our laboratory, immunization with CnaA alone or in combination with other toxoids/virulence factors conferred partial protection against virulent *C. perfringens* challenge [25].

The present study investigated the capability of collagen binding and *cnaA* gene level among the virulent and less virulent clinical *C. perfringens* isolates and evaluated any association between the capability to bind to collagen types I–V/gelatin and NE pathogenicity in chickens. For simplicity, three *C. perfringens* groups were assigned: *netB^−^tpeL^−^*, *netB^+^tpeL^−^,* and *netB^+^tpeL^+^*. The *netB^−^tpeL^−^* group also contained the avirulent isolates (such as N10 and SM101, demonstrated by *Eimeria maxima*/*C. perfringens* challenge studies) and clinical NE strains. Our results showed that the avirulent isolates (N10 and SM101, *netB^−^*) demonstrated a weak binding activity to collagen types III, V, and gelatin compared to the other virulent isolates in the same group or other group. There were variable capabilities of binding to these collagens among different groups. Interestingly, the *netB^−^tpeL^−^* group had statistically significant capabilities of binding to collagen II than the *netB^+^tpeL^−^* and *netB^+^tpeL^+^* groups. The reason why the *netB^−^ C. perfringens* isolates bind more potently to collagen II than *netB^+^* isolates (*netB^+^tpeL^−^* and *netB^+^tpeL^+^*) is not completely understood. The lower binding activities to collagenase type II in *C. perfringens* type G isolates may be associated with a high frequency of collagenase variant type II gene fragments that encode a truncated collagenase with the missing N-terminal part of the collagenase unit [26]. The absence of the NetB toxin in *netB^−^* isolates may lead to the upregulation of the production of the collagen II binding protein that compensates for the loss of NetB or less interference with their binding to collagen II. Interestingly, among the eight isolates that showed the potent binding capability to collagen III (OD562 ≥ 1.0), five out of eight isolates came from *netB^+^tpeL^+^* isolates. All five *netB^+^tpeL^+^* isolates showed statistically significant potent capabilities of binding to collagen III than the *tpeL^−^* (*netB^+^tpeL^−^* and *netB^−^tpeL^−^*) groups. This unique characteristic may be related to the presence of the *tpeL^+^* gene. The TpeL protein, a member of the large clostridial toxin (LCT) family [27,28], is reported to promote the disease severity of NE in chickens [3,15], while NetB is a pore-forming toxin that has been shown to disrupt the integrity of cell membranes [4]. The presence of both TpeL and NetB toxins may cause more severe tissue damage and superior binding to collagen type III. The isolate *netB^+^tpeL^+^* tpeL15 was the outstanding isolate that firmly bound to all collagen types and gelatin. In our previous genomic study [17], *netB^+^tpeL^+^* isolate tpeL17 shared the same fibronectin-binding protein with other strains (Del1, N11, SM101, Strain 13), and shared the same fimbrial adhesion protein with other virulent isolates (Del1 and N11 isolates). The reason why the *netB^+^tpeL^+^ C. perfringens* isolates potently bound to collagen type III merits further investigation.

Adhesin CnaA protein may play an important role in bacterial interaction with collagens and colonization, and our PCR and qPCR results demonstrated that more copies of *cnaA* gene were readily detected in a majority of the *netB^+^* virulent isolates (*netB^+^tpeL^−^* and *netB^+^ tpeL^+^*), but much less in *netB^−^tpeL^−^* isolates. The association of higher copies of the *cnaA* gene with *netB^+^* strains suggested that the product encoded by this *cnaA* gene may have a synergistic effect with NetB toxin in the NE pathogenesis. Furthermore, the sandwich ELISA assay in another pilot study using CnaA-specific monoclonal antibodies enabled us to detect *cnaA* gene expression at the protein level in various *C. perfringens* overnight culture supernatants [29], especially in few clinical virulent isolates, including the well-characterized pathogenic N11 (28.5 ng/mL), Del1 (20.1 ng/mL), and tpeL17 (6.6 ng/mL) isolates. N11, a *netB^−^tpeL^−^* strain isolated from healthy chicken gut from our previous study, induced necro-hemorrhagic enteritis lesions in combination with *Eimeria maxima* pre-exposure [16,30,31]. It appears that the concentrations of CnaA proteins in the supernatant of these pathogenic *C. perfringens* isolates may be associated with the bacterial growth rate (N11 > Del1 > tpeL17) as well, and the generation of the higher levels of toxins (for example, NetB and TpeL secreted by tpeL 17 isolate) may slow down the growth rate, therefore affecting the CnaA production in the supernatant (unpublished data). Further investigations may be warranted to better define the complex association among growth rate, toxin productions, genotypes, and pathogenicity. The collagen adhesin-like proteins, as an important virulence factor also developed by many other pathogenic Gram-positive bacteria to promote bacteria–host interaction [32,33], may be useful targets utilized to control multiple bacterial infections at the critical steps of initiation and persistence.

## 5. Conclusions

We have demonstrated that clinical isolates of *C. perfringens* are capable of binding to collagen types I and III-V, compared to avirulent isolates. The *netB^+^tpeL^+^* isolates potently bound to collagen type III. All the collection isolates carrying the *cnaA* gene with higher copy numbers were mainly *netB^+^* isolates. The collagen adhesin *cnaA* gene plays an important role in the adherence of *C. perfringens* to collagen types during the bacterial colonization stage in poultry. Our results suggest that the collagen binding capability correlates positively with their NE pathogenicity levels, especially for *C. perfringens* isolates carrying crucial virulence factors and virulence-associate factors such as NetB, CnaA, and TpeL.

## Figures and Tables

**Table 2:** Primer sequences and target genes used in the qRT–PCR analysis.

Target Gene	Primer Sequences (5’-3’)
16S rRNA	F-GGGGGTTTCAACACCTCC
	R-GCAAGGGATATCAAGTGT
cnaA	F-GGTGGATGGGCAACATTTAC
	R-CCTTGCTTGGATTCACCAGT

**Table 3:** Results of comparison of *C. perfringens* isolates for *cnaA* gene detection by PCR and qPCR.

GENOTYPE	*netB^−^tpeL^−^*									*netB^−^tpeL^+^*				
ISOLATE ID	13	CP1	CP10	CP15	CP23	JGS	N10	N11	SM101	C11	tpeL15	tpeL17	tpeL18	tpeL19
***cnaA* PCR**	+	+	+	+	+	+	+	+	+	++	++	++	++	++
***cnaA* qPCR**	−	−	−	−	−	−	−	−	−	+++	+++	+++	+++	+++
**GENOTYPE**	***netB^+^tpeL^+^***													
**ISOLATE ID**	C1	C2	C3	C5	C6	C7	C8	C9	CP10	CP12	CP13	CP14	CP16	Del 1
***cnaA* PCR**	++	++	++	++	++	++	++	+	++	++	++	+	+	+
***cnaA* qPCR**	+++	+++	+++	+++	+++	+++	+++	−	+++	+++	+++	−	−	+/−

Note: +++ (strong expression), ++ (intermediate expression), + (weak expression), − (very weak or no expression).

## Data Availability

The data presented in this study are available within the article.

## Abbreviations

CMG = chopped meat glucose; CP = Clostridium perfringens; NE = necrotic enteritis; *netB* = necrotic enteritis toxin B-like gene; *tpeL* = Clostridium perfringens lethal toxin gene; *cnaA* = adhesin protein can gene; OD = optical density; qPCR = quantitative polymerase chain reaction.
